# Small Phenolic Metabolites at the Nexus of Nutrient Transport and Energy Metabolism

**DOI:** 10.3390/molecules30051026

**Published:** 2025-02-24

**Authors:** Reham Mhawish, Slavko Komarnytsky

**Affiliations:** 1 Plants for Human Health Institute, North Carolina State University, 600 Laureate Way, Kannapolis, NC 28081, USA; rmmhawis@ncsu.edu; 2 Department of Food, Bioprocessing, and Nutrition Sciences, North Carolina State University, 400 Dan Allen Drive, Raleigh, NC 27695, USA; 3 Department of Nutrition and Food Technology, Jordan University of Science and Technology, P.O. Box 3030, Irbid 22110, Jordan

**Keywords:** phenolic acids, polyphenols, glycemic control, energy metabolism, metabolic adaptability, insulin resistance, nutrient signaling pathways, nutritional biochemistry

## Abstract

Over time, human metabolism evolved to accommodate the challenges and benefits of plant foods that contain high amounts of carbohydrates and polyphenols. The latter are typically metabolized into small phenolic metabolites, including phenolic acids and their endogenous and microbial derivatives, that influence interconnected metabolic pathways involved in nutrient transport, energy metabolism, and neurotransmitter balance. Unlike other natural products, their biological effects arise from weak interactions with multiple molecular pathways rather than a single high-affinity receptor, making them versatile regulators of metabolic health. These compounds also modulate glucose transporters and carbohydrate metabolism, playing a crucial role in postprandial glucose and insulin responses. This review addresses the critical role of phenolic metabolites in metabolic health, with a focus on glucose homeostasis, insulin sensitivity, and carbohydrate metabolism. Incorporating polyphenols and phenolic acids into dietary strategies offers significant potential for improving insulin sensitivity, reducing metabolic disorder risks, and promoting whole-body glucose homeostasis. Furthermore, understanding how phenolic metabolites interact with metabolic pathways is essential for developing future effective nutritional strategies to support metabolic health.

## 1. Introduction

The variability in diets among early hominids underscores the importance of ecological flexibility rather than exclusive reliance on a specific type of food [1]. While the increased consumption of meat may have contributed to shorter digestive tracts—freeing up metabolic energy to support brain development—and enabled complex cognitive abilities by providing essential nutrients such as iron, zinc, and vitamin B12, it was the continuous inclusion of diverse plant-based foods that enabled survival and persistence despite ever-changing ecological and social pressures [2]. *Australopithecus* from the 3.5-million-year-old Sterkfontein site in South Africa ate a varied plant-based diet that was broadly vegan [3]. Proteins from green leaves, though relatively low compared to animal sources, can still contribute essential amino acids when consumed in sufficient quantities, and polyphenols are an inevitable component of plant-based diets [4]. Over time, the ability of humans and other species to metabolize or tolerate polyphenols likely co-evolved with their reliance on plant-based foods, reflecting a balance between challenges and benefits in plant consumption [5].

Plant small phenolic metabolites, including phenolic acids, phenolic acid esters, as well as endogenous and microbial catabolites of flavonoids, chalcones, stilbenes, lignans, and tannins (loosely defined as polyphenols), represent a promising nexus in the regulation of interconnected metabolic pathways [6]. These small molecules exert pleiotropic effects across many biochemical networks [7], including nutrient transport [8], energy metabolism [9], immune regulation [10], and neurotransmitter synthesis [11], making them ideal candidates for redefining healthy metabolic states.

Unlike many other natural products from plants, small phenolic metabolites do not have a common receptor or a primary molecular target in the human body, with the possible exception of bitter taste chemoreceptors (TAS2Rs) [12]. Instead, their biological effects arise from interactions with multiple molecular pathways and cellular processes. These metabolites are well-known for neutralizing free radicals and reducing oxidative stress [13], a function that is not mediated by a specific receptor but occurs through direct chemical interactions with reactive oxygen species. Additionally, phenolic metabolites modulate cell signaling pathways involved in energy metabolism (e.g., AMP-activated protein kinase), immune function (e.g., nuclear factor kappa B), and beta-estrogen receptor signaling [14]. However, these effects are achieved through a combination of weak interactions and broader regulatory mechanisms rather than a single, high-affinity target.

Small phenolic metabolites also share striking similarities with certain endogenous biochemical pathways, including those involving serotonin, dopamine, and catecholamines [15], as well as direct conjugation to amino acids in mitochondria as a part of the phenolic detoxification and nitrogen deportation systems [16]. These similarities arise from their structural features, such as hydroxyl groups attached to aromatic rings, which enable them to interact with similar enzymes, transporters, and metabolic systems. This dual role in mimicking endogenous pathways and engaging in cellular detoxification underscores the unique and versatile nature of small phenolic metabolites in human physiology. In this review, we therefore aim to highlight the complex roles of small phenolic metabolites, focusing on their ability to influence metabolic pathways and enhance resilience, as well as act as modulators of nutrient transport, energy metabolism, mitochondrial function, and neurotransmitter balance.

## 2. Baseline Phenolic Acid Metabolites in Humans

### 2.1. Blood and Urine

Baseline phenolic acid metabolites in humans are derived from the endogenous metabolic pathways and are heavily influenced by dietary polyphenols, serving as important markers of metabolic health and healthy gut microbiome [17]. These metabolites, including four major series of benzoic, phenylacetic, phenylpropanoic, and cinnamic acids, are commonly found in blood, urine, and feces in different quantities, reflecting their absorption, metabolism, and excretion [18].

In blood, phenolic acids typically exist in conjugated forms such as glucuronides, sulfates, or methylated derivatives, as they undergo extensive phase II metabolism in the liver [19]. Blood phenolic acid concentrations are typically lower compared to urine due to rapid metabolism and excretion [20].

In urine, phenolic acids are highly abundant, as they represent a primary route of excretion for systemically metabolized and gut-derived compounds, with phenylacetic acid, 4-hydroxyphenylacetic acid, and hippuric acids often being the predominant waste metabolites [21]. As such, these metabolites show low to no biological activity when compared with other series of phenolic acids [22]. Higher levels of urinary phenolic acids are often observed in individuals with a plant-rich diet, while lower levels may indicate metabolic dysfunction or poor gut microbial diversity.

**Figure 1:**
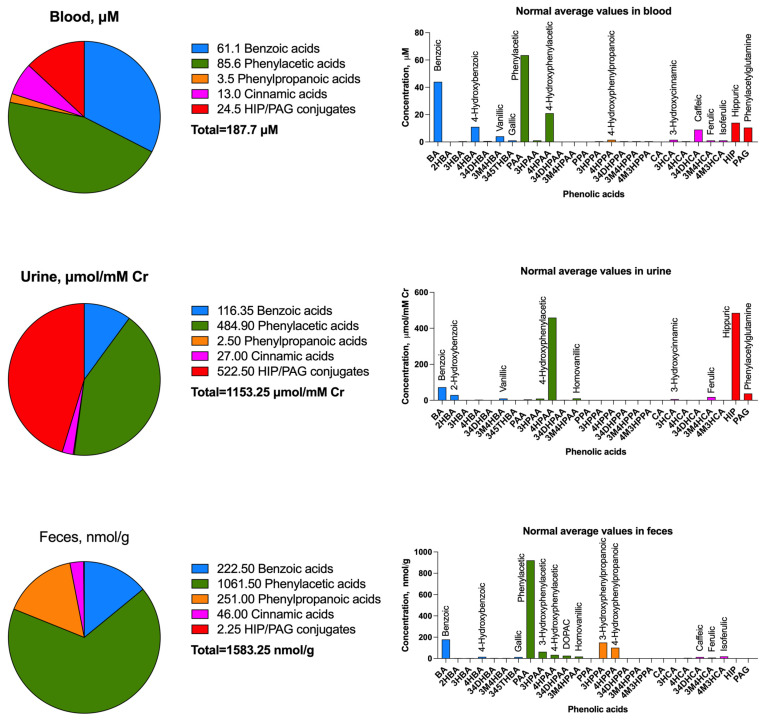
Major phenolic acids found in human blood (μM), urine (μmol/mM creatinine), and feces (nmol/g wet weight). Normal levels of each phenolic acid were retrieved and averaged across multiple clinical studies summarized in the Human Metabolome database [23]. Benzoic acids (blue) included benzoic acid (BA), 2-hydroxybenzoic acid (2HBA), 3-hydroxybenzoic acid (3HBA), 4-hydroxybenzoic acid (4HBA), 3,4-dihydroxybenzoic acid (3,4DHBA, protocatechuic acid), 3-methoxy-4-hydroxybenzoic acid (3M4HBA, vanillic acid), and 3,4,5-trihydroxybenzoic acid (345THBA, gallic acid). Phenylacetic acids (green) included phenylacetic acid (PAA), 3-hydroxyphenylacetic acid (3HPAA), 4-hydroxyphenylacetic acid (4HPAA), 3,4-dihydroxyphenylacetic acid (3,4DHPAA, DOPAC), and 3-methoxy-4-hydroxyphenylacetic acid (3M4HPAA, homovanillic acid). Phenylpropanoic acids (orange) included phenylpropanoic acid (PPA), 3-hydroxyphenylpropanoic acid (3HPPA), 4-hydroxyphenylpropanoic acid (4HPPA, desaminotyrosine), 3,4-dihydroxyphenylpropanoic acid (3,4DHPPA, dihydrocaffeic acid), and 3-methoxy-4-hydroxyphenylpropanoic acid (3M4HPPA, dihydroferulic acid). Cinnamic acids (pink) included cinnamic acid (CA), 3-hydroxycinnamic acid (3HCA, m-coumaric acid), 4-hydroxycinnamic acid (4HCA, p-coumaric acid), 3,4-dihydroxycinnamic acid (3,4DHCA, caffeic acid), 3-methoxy-4-hydroxycinnamic acid (3M4HCA, ferulic acid), and 4-methoxy-3-hydroxycinnamic acid (4M3HCA, isoferulic acid). Amino acid conjugates (red) included those bound to glycine (hippuric acid) and glutamine (phenylacetylglutamine).

Blood profiles are typically dominated by the benzoic and phenylacetic acids, their respective 4-hydroxy metabolites, as well as their glycine and glutamine conjugates. Most benzoic acids are rapidly metabolized into hippuric acid and its derivatives prior to their accumulation in urine, while the bulk of phenylacetic acids are converted into 4-hydroxyphenylacetic acid and are also targeted for renal excretion. Their methylated derivatives, including vanillic acid, homovanillic acid, ferulic acid, isoferulic acid, and dihydroferulic acid. These are found in baseline circulation in small quantities due to their increased hydrophobicity and affinity for other human tissues. Nearly all phenylpropanoic acids in human circulation are derived from microbial transformation of other phenolic acid metabolites in the gut, the primary site of their abundance (Figure 1).

### 2.2. Feces and Microbial Biotransformation

Fecal phenolic profiles also vary widely depending on gut microbiota diversity, fiber and polyphenol intake, as well as colonic transit time [24]. Fecal phenolic acid patterns can also indicate microbial dysbiosis, which has been associated with chronic diseases such as obesity and inflammatory bowel disease [25]. On the other hand, different classes of dietary polyphenols are often metabolized in different subsets of phenolic acid metabolites [6], based on their core phenolic structure and the microbial profiles of the host microbiome. Interindividual variability in these metabolites therefore highlights the complex interplay among diet, host metabolism, and gut microbiota and will be discussed in more detail in Section 3.

Of significant interest is the presence of gut-derived phenylpropanoid acid metabolites in circulation despite their hydrophobicity, especially the 3-hydroxyphenylpropanoic (m-dihydrocoumaric acid) and 4-hydroxyphenylpropanoic acid (p-dihydrocoumaric acid, desaminotyrosine) [23], as well as 3-methoxy-4-hydroxyphenylpropanoic acid (3M4HPPA, dihydroferulic acid) [22]. Tissues near the gastrointestinal lumen, such as gut epithelial and smooth muscle cells, as well as blood vessel cells, are likely exposed to higher levels of polar microbial catabolites from dietary fiber and polyphenols. In contrast, dihydro- and methylated phenolic metabolites are better suited to reach distant metabolically active tissues, where they may exert biological effects [22]. The unexplored structure–activity relationships of phenolic metabolites, influenced by methylation and hydrophobicity, are critically lacking to explain their differing capacities for membrane permeation and molecular interactions.

## 3. Phenolic Acids Released from Dietary Polyphenols

Polyphenols is a major group of plant secondary metabolites found throughout plant tissues, collectively forming thousands of distinct chemical structures characterized by hydroxylated aromatic rings [26]. The estimates for daily total polyphenol intake vary across the country and date of the study, but it is reasonable to assume the upper limit of intake of 1370 mg/day total polyphenols among coffee and tea consumers, and a 540 mg/day intake in people who do not consume these drinks regularly, as described in a recent 77,441 participant study [27]. Fruits (apples, oranges), vegetables (onions, spinach, lettuce), cocoa products, and wine were the major other sources, with additional contributions from potatoes, cereals, legumes, or berries depending on the geographical region. The mean overall intakes were summarized as 910 mg total polyphenols, including 360 mg of total flavonoids and 410 mg of total phenolic acids [28].

Stomach absorption of intact polyphenols is minimal, but passive absorption of aglycones can occur in the upper gastrointestinal tract after enzymatic hydrolysis by lactase-phloridzin hydrolase and β-glucosidases in the small intestine brush border [29]. Once absorbed, polyphenols undergo phase I (oxidation, reduction) and phase II (conjugation) metabolism to convert less-polar molecules into water-soluble metabolites for renal excretion. In humans, phase II conjugation (glucuronidation in the endoplasmatic reticulum, sulfation in the cytoplasm, methylation in both the cytoplasm and ER, and conjugation with amino acids in mitochondria) predominates over phase I metabolism [30]. The degree of phase II conjugation depends on the physiochemical properties of phenolics, with diverse fragmentation patterns creating distinct metabolic signatures of benzoic and cinnamic acid metabolites [6]. In turn, phenylacetic and phenylpropanoic acids have no significant dietary sources but are derived from colonic microbial metabolism of dietary polyphenols [18]. Microbial transformation of caffeoylquinic acids produces large quantities of dihydrocinnamic acids that also enter circulation [31]. Beyond detoxification, phase II metabolism of dietary phenolic metabolites plays a critical role in regulating physiological levels of amino acid precursors (glycine, glutamine), supporting mitochondrial energy metabolism, and nitrogen waste deportation [32].

**Figure 2:**
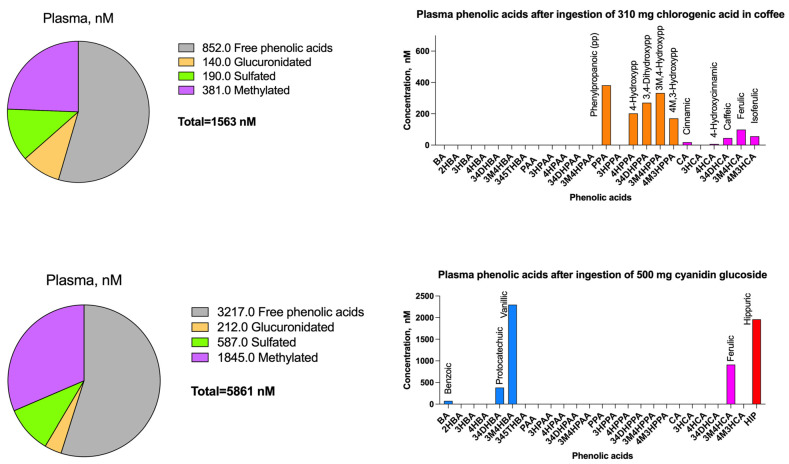
Major phenolic acids found in human blood (nM) after ingestion of 310 mg of chlorogenic acid in coffee (**top**) and 500 mg cyanidin glucoside (**bottom**), after [33,35]. The nomenclature of phenolic acid metabolites is listed in Figure 1.

A study on healthy volunteers consuming coffee containing 310 mg of chlorogenic acid identified 56 phenolic metabolites in plasma [33]. Caffeic acid metabolites were primarily sulfated, ferulic acids were equally glucuronidated and sulfated, and coumaric acids were present in small amounts, while dihydro metabolites, likely formed by gut microbiota, appeared mainly in free forms (Figure 2, top panel). The study highlighted a shift from caffeic acid derivatives to ferulic and isoferulic metabolites in plasma, mediated by catechol-O-methyltransferase (COMT), and likely omitted the presence of downstream benzoic acid metabolites such as vanillic and 3-hydroxybenzoic acids, possibly resulting from partial endogenous β-oxidation as shown previously [34].

A study on eight healthy male volunteers consuming 500 mg of 13C-labeled cyanidin-3-glucoside revealed extensive phase II metabolism and microbial biotransformation. Free, methylated, and glucuronidated forms peaked at 760 nM within 1–2 h post-bolus [35]. Vanillic acid was the dominant plasma and urine metabolite, surpassing protocatechuic acid, with both preferring sulfation over glucuronidation. Hydroxycinnamic metabolites, such as caffeic acid and ferulic acid, were found in smaller amounts, with ferulic acid consistently dominating, indicating significant methylation of phenolic substrates. Ring fission reactions of anthocyanins appear to favor the production of benzoic acid metabolites, although this requires further investigation (Figure 2, bottom panel).

Flavanol breakdown in the form of epicatechin resulted in phase II metabolites, predominantly undergoing methylation and sulfation, with γ-valerolactones as the dominant group of compounds [36]. The breakdown pathway from γ-valerolactones to hippuric acid may involve phenylhydracrylic intermediates. Additionally, there appears to be a trend toward forming phenylacetic acids, hydracrylic acids, and flavandiols from flavonols [37], flavanones [38], and isoflavones [39], respectively, highlighting gaps in understanding the differences in metabolism of small phenolic metabolites from diverse parent compounds.

An additional source of variation in phenolic metabolites in circulation is their affinity for binding human serum albumin (HSA), which increases with the number of free aromatic hydroxyl groups [40]. HSA, the most abundant protein in human plasma, contains two binding sites for small organic molecules, including most phenolic acids [41]. Binding to HSA regulates the free, active concentration of a metabolite in the blood, and methylation of aromatic hydroxyls typically decreases this binding, potentially increasing the free fraction of methylated metabolites (methylated sink), enhancing their potency and tissue distribution. This is demonstrated by the detection of ferulic acid, but not caffeic acid, in blood cells after ingesting a polyphenol-rich extract, despite both being present in serum [42]. Deficiency in catechol-o-methyltransferase activity was preliminarily linked to disruption of glucose homeostasis in an animal model [43].

## 4. Overlap with Amino Acid Metabolism

Phenolic acid metabolism in humans shares several key pathways and intermediates with amino acid metabolism, highlighting their interconnected roles in health and physiology. Both phenolic acids and amino acids undergo biotransformation by gut microbiota, producing bioactive metabolites that influence systemic functions, such as neurotransmission and energy metabolism [44]. Enzymatic processes, including transamination and decarboxylation, are common to both metabolic pathways. These pathways also intersect in cellular antioxidant systems, where phenolic acids and amino acid-derived metabolites, such as glutathione, contribute to redox homeostasis [45]. Furthermore, phenolic acids can modulate the metabolism of aromatic amino acids, such as tryptophan, influencing pathways linked to serotonin production and immune regulation [46].

Phenylalanine is metabolized into phenylacetic acid, a key metabolite derived from its catabolic pathway. This occurs primarily through deamination to produce phenylpyruvic acid, which is further metabolized to phenylacetic acid [47]. This pathway is part of phenylalanine catabolism in humans and is particularly active in conditions like phenylketonuria, where a deficiency in the enzyme phenylalanine hydroxylase disrupts the conversion of phenylalanine to tyrosine, leading to the accumulation of phenylacetic acid and its derivatives [48].

Tyrosine is metabolized into p-hydroxyphenylacetic acid as a primary phenolic acid metabolite. This occurs through deamination of tyrosine to form p-hydroxyphenylpyruvic acid, which is further converted into p-hydroxyphenylacetic acid [49]. Additionally, tyrosine can also be metabolized into homovanillic acid (HVA) via the dopamine pathway, depending on its involvement in neurotransmitter metabolism [50]. These metabolites play significant roles in both physiological processes and diagnostic assessments of metabolic or neurological disorders.

Tryptophan is metabolized into indole-3-acetic acid (IAA), a primary phenolic acid derivative. This occurs via the microbial metabolism of tryptophan in the gut, where it is converted to indole by bacterial enzymes and subsequently oxidized to indole-3-acetic acid [51]. Indole-3-acetic acid and related metabolites are involved in various physiological and signaling processes, including gut–microbiota interactions. Additionally, tryptophan can be metabolized into kynurenic acid and other metabolites through the kynurenine pathway [52].

**Figure 3:**
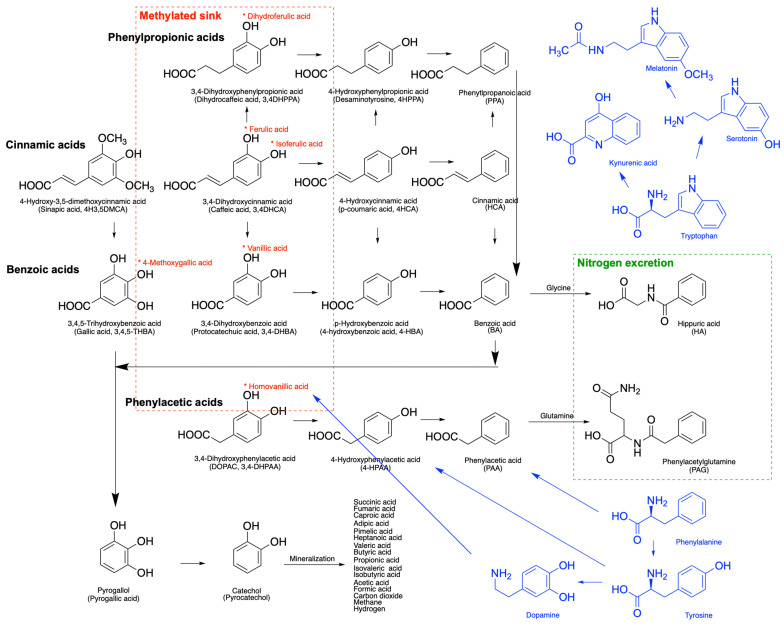
Major phenolic acid metabolites generated by host metabolism, as well as degradation and microbial fermentation of dietary polyphenols towards conjugation with amino acids (glycine and glutamine) and mineralization into small organic acids on the way to carbon dioxide, methane, or hydrogen production. The nomenclature of phenolic acid metabolites is listed in Figure 1. The red asterisk (*) denotes methylation sites and the respective names of the methylated phenolic metabolites. The red dashed area identifies these metabolites as a possible methylated reservoir of these compounds in human tissues. The green dashed area denotes two major metabolites responsible for nitrogen excretion. Blue color denotes aromatic amino acids and their metabolites.

The interconnected metabolic pathways of phenolic acids and aromatic amino acids provide a fascinating framework for understanding the health-promoting effects of polyphenols. The nitrogen deportation system, essential for nitrogen balance, is closely linked to these metabolic interactions. Compounds like hippuric acid and phenylacetylglutamine (PAG) serve as important mediators of nitrogen excretion. Phenolic metabolites can influence the nitrogen deportation system by modulating the microbial enzymes involved in aromatic amino acid catabolism, thereby altering the production of these substrates (Figure 3).

## 5. Energy Transport and Metabolism

### 5.1. Competitive Interactions with Organic Anion Transporters

Endogenous esterases, found in pancreatic secretions, brush border layers of the small intestine, and colonic microbiota, efficiently release phenolic metabolites from their parent compounds to facilitate absorption [53]. Most phase II metabolism, including glucuronidation, sulfation, and methylation, occurs in the enterocyte and liver, while processes like demethylation and dehydrogenation are restricted to the liver, establishing it as a key regulator of methylated phenolic metabolite levels [54]. Metabolites conjugated with a larger glucuronic acid moiety are more likely to enter enterohepatic circulation compared to sulfated or methylated derivatives, explaining their lower abundance in circulation. Absorption of phenolic metabolites from the gastrointestinal tract increases with hydrophobicity, while more polar structures are often methylated to improve uptake via monocarboxylate transporter MCT1 (SLC16A1) and transferred to the blood through MCT4 (SLC16A3) [55].

Urinary excretion of phenolic metabolites is regulated by another set of organic anion transporters (OATs) in the proximal kidney tubules. Different transporters, such as OAT1 (SLC22A6), OAT2 (SLC22A7), OAT3 (SLC22A8), and OAT4 (SLC22A11), exhibit variable affinities for free, glucuronidated, and sulfated metabolites [56]. Phenolic acids can also enter the endoplasmic reticulum (ER) through ATP-binding cassette (ABC) transporters like ABCG2, which play a role in regulating the availability of these bioactive compounds for further processing and secretion [57]. In mitochondria, phenolic acids enhance antioxidant defenses by supporting glutathione metabolism. They can interact with SLC7A11, the cystine/glutamate antiporter, to regulate the availability of cysteine for glutathione synthesis, indirectly impacting mitochondrial redox homeostasis [58]. Finally, SLC25A1, a citrate carrier in the mitochondrial inner membrane, may indirectly facilitate the trafficking of phenolic metabolites that interact with citrate metabolism [59]. These transport systems play a pivotal role in determining the levels of phenolic metabolites in cells and determine their ultimate routes of excretion.

### 5.2. Glucose Absorption in the Gut

Phenolic acids interact with glucose transporters in the gut and other tissues, influencing glucose absorption and cellular uptake. Two primary transporter families are involved are sodium–glucose co-transporters (SGLTs) and facilitative glucose transporters (GLUTs). Phenolic acids can modulate these transporters either directly by binding to them or indirectly by altering their expression and activity [60].

SGLT1, found in the small intestine, facilitates active glucose transport into enterocytes. Phenolic acids can inhibit SGLT1 activity, thereby reducing glucose absorption and dampening postprandial blood glucose spikes [8]. Phenolic acids have been shown to reduce glucose transport via SGLT1 and GLUT2-mediated glucose absorption, particularly when GLUT2 translocates to the brush border membrane of enterocytes in response to high glucose concentrations [61]. Phenolic acids interact with glucose transporters through various physicochemical mechanisms, including direct binding, modulation of transporter expression, and alteration of membrane dynamics. Studies suggest that certain phenolic acids can inhibit SGLT1-mediated glucose uptake by competing for binding sites or altering transporter conformation [62]. An extensive discussion on these effects and their application to glucose consumption was published recently [8]. Further research is needed to elucidate the structural basis of these interactions, the specificity of different phenolic acid derivatives for glucose transporters, and their dose-dependent effects in physiological conditions.

In peripheral tissues, phenolic acids influence GLUT4, a key transporter responsible for insulin-stimulated glucose uptake in muscle and adipose tissue. Phenolic acid metabolites, such as ferulic acid and its derivatives, have been found to modulate GLUT4 translocation to the plasma membrane, improving glucose utilization and insulin sensitivity [63]. This mechanism is particularly relevant for managing insulin resistance and type 2 diabetes mellitus. Moreover, phenolic acids can indirectly affect glucose transporters by either altering gut microbiota metabolism or directly modulating the activity of the bitter taste receptors [5]. In animal models, gastrointestinal cells co-express the bitter receptor mTAS2R108, the glucagon-like peptide-1 (GLP-1), and the GLP-1R receptor, and their expression levels are upregulated in response to bitter substances [12]. Model bitter substances also increase intracellular [Ca2+] in neuroendocrine STC-1 cells, a recognized model for glucose absorption and gastrointestinal hormone secretion [64], highlighting their potential for modulating glucose absorption and hormone secretion. These interactions contribute to the beneficial effects of phenolic acids on glycemic control, insulin sensitivity, and metabolic health.

### 5.3. Energy Metabolism and Insulin Resistance

Beyond direct effects on glucose transport, phenolic acids also modulate signaling pathways that regulate transporter activity. For instance, phenolic acids can activate AMP-activated protein kinase (AMPK), a critical regulator of GLUT4 expression and glucose metabolism [65]. Activation of AMPK by phenolic acids has been linked to reduced hepatic glucose production and enhanced glucose uptake in peripheral tissues [66]. Similar effects can also modulate insulin secretion, glucose release from the liver, insulin receptor activation, and glucose uptake by insulin-sensitive tissues [67]. Additionally, they influence hepatic glucose output and other aspects of metabolism, demonstrating their potential in managing diabetes and obesity [68].

p-Coumaric acid, a key phenolic acid in plant foods, exhibits antioxidant, anti-inflammatory, and anticancer properties. It lowers blood glucose, total cholesterol, and triglycerides in diabetic models while protecting pancreatic β-cells from oxidative stress and improving hepatic glucose metabolism by enhancing hexokinase and glucose-6-phosphate dehydrogenase activity [69]. Additionally, its antioxidant effects reduce liver oxidative stress, alleviate diabetic nephropathy, and, when present in highland barley grain alongside procyanidin B1, enhance glucose uptake and insulin sensitivity to help combat insulin resistance [70].

Caffeic acid, found in various fruits, vegetables, and beverages, lowers plasma glucose and enhances insulin sensitivity [71]. It regulates blood glucose by inhibiting α-glucosidase and α-amylase, reducing postprandial hyperglycemia [72]. In rodent models, caffeic acid decreases body weight, visceral fat mass, and plasma lipid levels while promoting fatty acid oxidation and suppressing lipogenesis via AMP-activated protein kinase [73]. Ferulic acid, abundant in cereal grains and fruits, reduces insulin resistance by modulating inflammatory pathways such as JNK, ERK, and NFκB [74]. In diabetic rat models, ferulic acid improves insulin sensitivity, enhances hepatic glycogenesis, and inhibits gluconeogenesis [75], with additional benefits observed when combined with dietary fibers like arabinoxylan for improved glucose tolerance and intestinal health [76]. However, most of these findings stem from animal studies, highlighting the need for caution in translating these results to human contexts. Given the compelling preclinical evidence, it is critical to advance ferulic acid and other phenolic acids into clinical trials to fully realize their therapeutic potential in metabolic health management.

## 6. Conclusions

Phenolic acid metabolites in humans are shaped by both endogenous metabolic processes and dietary polyphenol intake, with distinct profiles observed in blood, urine, and feces. These metabolites act as valuable biomarkers of metabolic health, reflecting the interplay between diet and metabolism. They directly influence nutrient transport and energy metabolism, further emphasizing their role in maintaining postprandial glucose and insulin responses. While studies have identified key metabolites in blood, urine, and feces, significant gaps remain regarding their transport, tissue-specific distribution, and long-term metabolic effects. Future research should address these discrepancies, explore interindividual variability in phenolic acid metabolism, and establish standardized methodologies for assessing their role as biomarkers of metabolic health. This will allow for developing novel dietary strategies for improving insulin sensitivity and reducing the risk of metabolic disorders by modulating carbohydrate metabolism in the gut, central to the whole-body glucose homeostasis.

## Data Availability

Data are contained within the article.
